# Is Mindful Parenting Associated With Adolescents’ Emotional Eating? The Mediating Role of Adolescents’ Self-Compassion and Body Shame

**DOI:** 10.3389/fpsyg.2018.02004

**Published:** 2018-10-26

**Authors:** Maria João Gouveia, Maria Cristina Canavarro, Helena Moreira

**Affiliations:** Center for Research in Neuropsychology and Cognitive-Behavioral Intervention, Faculty of Psychology and Educational Sciences, University of Coimbra, Coimbra, Portugal

**Keywords:** mindful parenting, self-compassion, body shame, emotional eating, weight

## Abstract

This study aimed to explore whether parents’ mindful parenting skills were associated with adolescents’ emotional eating through adolescents’ levels of self-compassion and body shame. The sample included 572 dyads composed of a mother or a father and his/her child (12–18 years old), with normal weight (BMI = 5–85th percentile) or with overweight/obesity with or without nutritional treatment (BMI ≥ 85th percentile) according to the WHO Child Growth Standards. Parents completed self-report measures of mindful parenting (Interpersonal Mindfulness in Parenting Scale), and adolescents completed measures of self-compassion (Self-Compassion Scale-Short Form), body shame (Experience of Shame Scale), and emotional eating (Dutch Eating Behavior Questionnaire). Two path models, one with the total score for mindful parenting and the other with its dimensions, were tested in AMOS. Mindful parenting, specifically the dimension of compassion for the child, was indirectly associated with emotional eating through adolescents’ self-compassion (point estimate = −0.27, *p* = 0.03, CI 95% [−0.61, −0.06]) and through self-compassion and body shame sequentially (point estimate = −0.19, *p* = 0.03, CI 95% [−0.37, −0.05]). The path model was invariant across weight groups but not across adolescents’ sex (the indirect effects were significant among girls only). This study provides a novel comprehensive model of how mindful parenting, especially the dimension of compassion for the child, can be associated with adolescents’ emotional eating behaviors by suggesting a potential sequence of mechanisms that may explain this association. This study suggests the beneficial effect of both mindful parenting and adolescents’ self-compassion skills for adolescent girls struggling with feelings of body shame and emotional eating behaviors.

## Introduction

Adolescence is the key period for the emergence and development of body-related issues and disordered eating behaviors (e.g., Stice, 2002; Neumark-Sztainer et al., 2011; Helfert and Warschburger, 2013). Disordered eating behaviors, such as emotional eating, are considered serious public health concerns for youth since they are developmental pathways to obesity even after weight loss (Thayer, 2001; Braet et al., 2008; O’Reilly and Black, 2015; Eichen et al., 2017). Therefore, it is critical to understand psychosocial and emotional processes related to disordered eating behaviors (Stice, 2002; Neumark-Sztainer et al., 2011) and to identify protective psychological skills that can help youths develop a healthier relationship with their bodies and with food. Self-compassion has recently been suggested to have a beneficial role in body-related issues and in disordered eating (e.g., Braun et al., 2016; Rahimi-Ardabili et al., 2018). In addition, considering the important impact that parents have on psychological functioning and on the eating behavior of their children, it is also critical to understand how parenting may be related to adolescents’ psychological processes and disordered eating behaviors. Mindful parenting is a parenting approach that may promote adolescents’ self-compassion skills and psychological functioning (e.g., Parent et al., 2016; Moreira et al., 2018). Nevertheless, no studies have explored how mindful parenting may be associated with adolescents’ disordered eating behaviors.

Emotional eating is a disordered eating behavior characterized by eating as an avoidant coping strategy to temporarily alleviate negative emotional states (Braet et al., 2014). Therefore, this eating behavior is intrinsically related to the inability to adequately regulate emotional states, especially painful or stressful ones (Evers et al., 2010). Emotional eating has been associated with poor psychological adjustment and eating disorders (e.g., anxiety and depressive symptoms, bulimia, binge eating disorder; Braet et al., 2008; Goossens et al., 2009); it is also a very common denominator and antecedent of other disordered eating behaviors (e.g., overeating; Zeeck et al., 2011). Emotional eating is more frequent among adolescents than among children (Wardle et al., 1992; Pearson et al., 2012; Bennett et al., 2013) and more frequent among youths with overweight/obesity than among youths with normal weight (Braet and van Strien, 1997).

One factor that has been considered a significant risk factor for engagement in disordered eating behaviors, such as emotional eating, is having negative feelings about one’s body (Stice, 2002; Neumark-Sztainer et al., 2011). Although the literature has preferentially focused on body dissatisfaction issues among adolescents, especially those with overweight and obesity, there is also some interest in studying the role of body shame. Similar to body dissatisfaction, body shame involves negative thoughts and emotions about one’s body but also encompasses more general negative feelings about the self that is objectified on the basis of the appearance (Calogero, 2012). Therefore, body shame arises from the evaluation of oneself or the perception that others evaluate oneself as inferior, flawed, or unattractive, with a desire to hide oneself and one’s body (Gilbert, 2002).

To better understand this self-oriented emotion, one must consider the role of body image ideals (i.e., to be thin or muscular) strongly perpetuated by society that encourage weight stigma, which is highly prevalent among youths with overweight and obesity, especially among girls (Smolak and Levine, 2001; Latner and Stunkard, 2003; Puhl and Latner, 2007). Moreover, this weight-related stigmatization very often leads to generalizations about individuals with overweight/obesity, including negative generalizations such as having low intelligence and poor social skills (Lynagh et al., 2015). Adolescents (especially in the middle and late stages) are particularly vulnerable to the development of appearance-related issues in the pursuit of the ideal body perpetuated by society and the desire to be accepted by others (Stice, 2002; Neumark-Sztainer et al., 2011; Helfert and Warschburger, 2013). Therefore, the frequent stigmatization that adolescents with overweight/obesity face increases the risk for negative psychological outcomes including poor quality of life and higher levels of body shame or eating disorders (Stice, 2002; Puhl and Latner, 2007; Eisenberg et al., 2012), thus influencing the way adolescents feel and relate to themselves.

Despite the scarce literature regarding the role of body shame among adolescents with different weights, there is some evidence to support the negative effect that this self-conscious emotion has on psychological function (Moreira and Canavarro, 2017a) and on the eating behavior of youths, especially those with overweight/obesity (Mustapic et al., 2015; Iannaccone et al., 2016). For instance, body shame was found to mediate the relationship between self-esteem and eating disorders among adolescents with different weights (Iannaccone et al., 2016), between body dissatisfaction and eating behaviors among adolescent girls with different weights (Mustapic et al., 2015), and between dispositional mindfulness and quality of life among older adolescents with overweight and obesity (Moreira and Canavarro, 2017a). Overall, these studies suggest that the greater one’s body shame is, the higher the probability of having negative emotions about oneself or lower levels of positive psychological resources, thus leading to the development of disordered eating behaviors and psychological problems. Therefore, when adolescents experience higher levels of body shame, they may struggle to regulate those negative emotions about themselves and be more prone to engage in compensatory behaviors such as emotional eating.

Self-compassion is a psychological resource aimed at alleviating one’s suffering with a caring and nurturing mentality, and it has been considered an adaptive strategy of emotion regulation or a coping strategy (McBeth and Gumley, 2012; Sirois et al., 2015). Self-compassion can be broadly defined as an adaptive way of relating to oneself by adopting an attitude of kindness toward one’s difficult experiences with the desire to relieve one’s own suffering (Gilbert and Procter, 2006; Neff, 2009). This state of mind encompasses higher levels of self-kindness and mindful awareness and the recognition that all human beings share a common humanity while demonstrating lower levels of self-judgment, overidentification, and isolation (Neff, 2003, 2009). Therefore, self-compassion has been associated with several psychological benefits among adolescents (e.g., lower levels of depression and anxiety symptoms, higher levels of well-being, greater life satisfaction, and less perceived stress; Neff and McGehee, 2010; Bluth et al., 2017).

Recently, some studies, mainly among adult women with both normal weight and overweight, have shown that self-compassion can also play an important role in the adoption of healthier behaviors, thus decreasing engagement in disordered eating behaviors and preventing negative weight-related outcomes (i.e., body dissatisfaction, body shame; e.g., Ferreira et al., 2013; Braun et al., 2016; Muris, 2016; Rahimi-Ardabili et al., 2018). Based on previous studies, self-compassion might enable more adaptive emotion regulation strategies, such as less self-critical thoughts and less cognitive-behavioral avoidance (which often trigger disordered eating behaviors and body dissatisfaction), increasing acceptance and thereby facilitating healthy weight management (Adams and Leary, 2007; Mantzios and Wilson, 2014; Albertson et al., 2015). In addition, from a holistic point of view, a self-compassionate approach may simultaneously promote physiological and psychological self-care, that is, encouraging individuals to care equally about the body and the mind (Mantzios and Egan, 2017). Despite the growing interest in the field, to date, no studies have been conducted on the relationship between self-compassion skills and disordered eating behaviors among adolescents.

Parents can play an important role in the development of their children’s self-compassion skills (Moreira et al., 2018). Through a mindful parenting approach, parents encourage the non-judgmental acceptance of difficult emotional states and foster the use of adaptive strategies of emotion regulation, leading to better adjusted psychological outcomes (Townshend et al., 2016; McKee et al., 2017). Mindful parenting is a parenting style characterized by intentionally bringing mindful awareness to everyday parent-child interactions (Kabat-Zinn and Kabat-Zinn, 1997; Bögels and Restifo, 2015) through the following important parenting practices or skills: directing complete attention to the child and being fully present during parent–child interactions; adopting an attitude of compassion, sensitivity, and responsiveness toward the child; adopting an attitude of non-judgmental acceptance of the self as a parent and of the challenges of parenting; self-regulating parents’ own emotions and behaviors in the parent–child relationship in accordance with parenting values and goals; and developing emotional awareness of the self and the child (de Bruin et al., 2014; Moreira and Canavarro, 2017b).

Among the extensive research on this topic, studies have shown that parents with higher levels of mindful parenting adopt more positive parenting styles and practices and demonstrate more positive interactions and communication with their children (e.g., Lippold et al., 2015; Gouveia et al., 2016; Parent et al., 2016). Moreover, this parenting approach has been associated with several indicators of positive psychological functioning in children, such as lower levels of depressive, anxiety, internalizing and externalizing symptoms, and increased well-being (e.g., Bögels et al., 2013; Parent et al., 2016; Moreira et al., 2018). However, whether this parenting approach can help adolescents in their relationship with their body and eating behavior remains to be investigated. Nevertheless, it can be hypothesized that a parenting context based on mindful awareness, acceptance, and compassion toward the child may lead parents to more easily detect negative emotional states in their children and foster their expression, which can in turn facilitate adaptive emotion regulation of children’s internal states. Moreover, when parents adopt a compassionate stance toward their children, children may learn to accept themselves as they are and as imperfect human beings. Therefore, in such a parenting context, adolescents may develop a healthier relationship with themselves, their bodies, and their eating behavior.

Research on the mechanisms that may account for the relationship between mindful parenting and adolescent outcomes is still in its infancy. Nevertheless, recent studies have suggested that psychological resources such as mindfulness and self-compassion skills may explain why mindful parenting plays a beneficial role in adolescents’ psychological functioning (Moreira et al., 2018; Wang et al., 2018). A better understanding of the mechanisms underlying the relationship between mindful parenting and adolescents’ eating behavior may enable the development of more tailored interventions for youths with disordered eating behaviors.

### The Present Study

The present study is a correlational, non-experimental, and cross-sectional study intended to explore whether mindful parenting is associated with adolescents’ emotional eating and whether this association is explained by adolescents’ self-compassion skills and body shame. These associations will be investigated in a group of adolescents with normal weight and adolescents with overweight/obesity. Although the relationship between mindful parenting and these outcomes has never been investigated, we hypothesize, based on previous studies regarding the role of mindful parenting on youth outcomes (e.g., Parent et al., 2016; Moreira et al., 2018), that higher levels of mindful parenting will be negatively associated with adolescents’ emotional eating through higher levels of adolescents’ self-compassion and lower levels of body shame.

Additionally, because these variables and/or the relationship between these variables may vary according to the stage of adolescence (Bluth et al., 2017), gender (Bluth et al., 2017), and weight group (Latzer and Stein, 2013), we also aimed to investigate whether the path model was invariant across two stages of adolescence (early vs. middle/late; Spano, 2004), gender (girls vs. boys), and three weight groups (normal weight vs. overweight/obesity not undergoing nutritional treatment vs. overweight/obesity undergoing nutritional treatment). We chose to take nutritional treatment into consideration because previous studies have generally found worse psychological outcomes among youth with overweight/obesity undergoing nutritional treatment than among youths with overweight/obesity from community samples (Goossens et al., 2009). In addition, since mindful parenting skills may vary according to parents’ gender (Medeiros et al., 2016) and since parental weight status may influence adolescent outcomes (Bahreynian et al., 2017), we aimed to analyze the invariance of the path model across parents’ gender (father vs. mother) and weight status (normal weight vs. overweight/obesity). We expect adolescents who are in the early stage of adolescence, boys, and adolescents with normal weight to report higher levels of self-compassion and lower levels of body shame and emotional eating than adolescents who are older, girls, and adolescents with overweight/obesity (e.g., Grabe et al., 2007; Braet et al., 2008; Bluth et al., 2017). We also expect fathers to report lower levels of mindful parenting skills than mothers (Medeiros et al., 2016). In addition, based on previous studies showing a stronger association between self-compassion and well-being outcomes among older adolescents (Bluth and Blanton, 2015) and girls (Moreira et al., 2018) and showing this association to be mediated by body shame (Moreira and Canavarro, 2017a), we expected to find stronger associations between adolescent outcomes among adolescents in the middle/late stage and among girls.

## Materials and Methods

### Participants

The sample comprised 572 dyads composed of a mother (*n* = 445; 77.8%) or a father (*n* = 127; 22.2%) and an adolescent between 12 and 18 years of age (*M* = 14.34, *SD* = 1.59). Of these adolescents, 323 had normal weight (56.5%; BMI = 3–85th percentiles), and 249 had overweight or obesity (43.5%; BMI ≥ 85th percentile; WHO, 2006) according to the WHO Child Growth Standards. To accomplish the purpose of this study, we used the following inclusion criteria: (1) age between 12 and 18 years old; (2) no serious mental illness, developmental delays or genetic syndromes for which obesity is a comorbidity (according to teachers/nutritionists and educational/medical files); and (3) ability to understand and answer the questionnaires (according to teachers/nutritionists and educational/medical files). The main sociodemographic and clinical characteristics of the sample are presented in Table 1.

**Table 1:** Parents’ and adolescents’ sociodemographic and clinical characteristics by weight groups and group differences.

	Adolescents with normal weight *n* = 323	Adolescents with overweight/obesity not undergoing nutritional treatment *n* = 110	Adolescents with overweight/obesity undergoing nutritional treatment *n* = 139	Group differences	
				*F/*χ^2^	$\eta_{p}^{2}/\Phi$
**Parents**			
Age (years) *M*(*SD*); range	44.16 (5.43); 31–61	43.32 (4.62); 31–56	43.32 (5.36); 30–58	1.77	0.006
Gender *n*(%)					
Male	120 (37.2)	4 (3.6)	3 (2.2)	96.07^∗∗∗^	0.368
Female	203 (62.8)	106 (96.4)	136 (97.8)		
Education level *n*(%)					
Basic or secondary	255 (78.9)	91 (82.7)	120 (86.3)	3.65	0.055
Graduate or post-graduate	68 (21.1)	19 (17.3)	19 (13.7)		
Area of residence *n*(%)					
Urban	76 (23.5)	21 (19.1)	40 (28.8)	3.24	0.021
Rural	247 (76.5)	89 (80.9)	99 (71.2)		
Cohabitation status *n*(%)					
Living with a partner	289 (89.5)	98 (89.1)	113 (81.3)	6.26^∗^	0.032
Not living with a partner	34 (10.5)	12 (10.9)	26 (18.7)		
Weight category *n*(%)					
Normal weight	141 (43.7)	40 (36.4)	32 (23.0)	17.74^∗∗∗^	0.100
Overweight/Obesity	182 (56.3)	70 (63.6)	107 (77.0)		
BMI *M*(*SD*); range	26.09 (3.94); 17.31–43.52	27.13 (4.60); 18.36–42.68	29.34 (5.37); 19.82–51.31	25.90^∗∗∗^	0.083
**Adolescents**					
Age (years) *M*(*SD*); range	14.27 (1.63); 12–18	13.88 (1.48); 12–18	14.85 (1.44); 12–18	12.51^∗∗∗^	0.042
Gender *n*(%)					
Male	119 (36.8)	54 (49.1)	61 (43.9)	5.77	0.100
Female	204 (63.2)	56 (50.9)	78 (56.1)		
zBMI *M*(*SD*); range	−0.17 (0.70); −1.93–1.00	1.70 (0.56); 1.01–3.56	2.20 (0.63); 1.04–3.95	759.11^∗∗∗^	0.727
Presence of Health Conditions *n*(%)					
Yes	82 (25.4)	28 (25.5)	91 (65.5)	74.10^∗∗∗^	0.097
No	241 (74.6)	82 (74.5)	48 (34.5)		
Type of health conditions *n*(%)					
Respiratory diseases	39 (47.6)	13 (46.4)	24 (26.4)	45.18^∗∗^	0.293
Metabolic diseases	0 (0.0)	1 (3.6)	9 (9.9)		
Neurologic diseases	3 (3.7)	4 (14.3)	6 (6.6)		
Heart diseases	9 (11.0)	1 (3.6)	13 (14.3)		
Mental diseases	18 (22.0)	6 (21.4)	23 (25.3)		
Dermatologic diseases	3 (3.7)	1 (3.6)	9 (9.9)		
Digestive system diseases	0 (0.0)	0 (0.0)	1 (1.1)		
Genetic diseases	3 (3.7)	0 (0.0)	0 (0.0)		
Kidney diseases	0 (0.0)	0 (0.0)	3 (3.3)		
Spinal diseases	2 (2.4)	0 (0.0)	3 (3.3)		
Oncologic diseases	0 (0.0)	1 (3.6)	0 (0.0)		
Others	5 (6.1)	1 (3.6)	0 (0.0)		

*^∗^p < 0.05; ^∗∗^p < 0.01; ^∗∗∗^p < 0.001.*

### Procedure

The sample was collected in three Portuguese public school units (*n* = 433) and three pediatric public hospitals (*n* = 139) in the central region of Portugal. Authorizations for sample collection were obtained from the Portuguese Data Protection Authority, the Ethics Committee of the Faculty of Psychology and Educational Sciences of the University of Coimbra, the Ethics Committee, and the Board of Directors of each hospital and school unit. All participants were informed of the voluntary nature of the study and the confidentiality and anonymity of their answers. Participation in the study occurred at a single time point and consisted of the completion of self-report questionnaires that took, on average, 25 min for the parents and 15 min for the adolescents. A protected, safe and supportive atmosphere was provided during the administration of the questionnaires, both in schools and in hospitals, to ensure the dignity and the privacy of the participants. All participants were instructed to remain silent while completing the questionnaires, unless they had any doubt or questions. Moreover, they were instructed, both orally and in the written instructions provided on the first page of the questionnaire, to answer individually and honestly.

Dyads collected from public schools were recruited between March 2015 and April 2016. In total, 91 classes from the three units were randomly selected to participate in the study. Each class was visited twice by a research assistant. The purpose of the first visit was to present the study and its aims and to give each adolescent an envelope containing a letter explaining the study, the parent’s informed consent form, and two identical questionnaires for the parents (one for the mother and one for the father). All parents completed a questionnaire with sociodemographic and clinical information about themselves and their children and a self-report measure of mindful parenting [Interpersonal Mindfulness in Parenting (IMP) Scale]. One week later, on a second visit, those adolescents who assented to participate and whose parents provided informed consent completed the questionnaires. Adolescents completed the questionnaires in the classroom during a period of the class reserved for this purpose in the presence of the class teacher and the research assistant, who could assist them whenever necessary.

Dyads from hospitals were recruited from nutrition outpatient services between June 2015 and November 2016. For adolescents who were undergoing nutritional treatment to lose weight, a nutritionist prescribed an adequate diet and scheduled physical activity and provided other behavior modification recommendations suited to each adolescent. Before or after the nutrition consultation, adolescents with overweight/obesity and their parents were approached by a research assistant who described the study and requested their participation. Those who agreed to participate provided verbal assent (adolescents) and informed consent (parents) and completed the questionnaires in a private consultation office provided for the purpose by the health institution, in the presence of the research assistant. If participants were not available to complete the questionnaires at that moment, they could complete the questionnaire at home, but were instructed to do so in a period of time reserved for that task and in a silent and comfortable atmosphere. Moreover, parents were instructed to help adolescents only if they had any doubt, but they were told not to influence adolescents’ answers. In such case, a preaddressed and stamped envelope was given to the participant to return the completed questionnaire by mail whenever possible. If questionnaires were not received in 2 weeks, a written message was sent to the mother or to the father to remind him/her to return the questionnaires.

Data from a total of 1532 mother/father–adolescent dyads were collected (1238 from schools and 294 from pediatric hospitals). Of these, 690 were triads composed of both a mother and a father of the same child. Therefore, 345 triads were randomly considered only as a mother–adolescent dyad, and the remaining 345 triads were considered only as a father-adolescent dyad to obtain a sample exclusively composed of independent observations (i.e., no father or mother was the parent of the same child). From the 1532 dyads, 912 cases were excluded because of non-responses to at least one study questionnaire or sociodemographic/clinical variables, and 48 cases were excluded for not meeting the inclusion criteria. These exclusions resulted in a final sample with 572 dyads composed of a mother or a father and an adolescent (*n* = 433 in the school sample; *n* = 139 in the hospital sample). Of the 433 dyads from public schools, 110 (25.40%) had a child with overweight/obesity, and 13 (11.82%) of them were undergoing nutritional treatment.

### Measures

#### Sociodemographic Information

Mothers, fathers, and adolescents self-reported their sociodemographic and clinical information (i.e., mothers and fathers: age, education level, area of residence, cohabitation status, weight, and height; adolescents: age, gender, weight, height, presence, and type of health conditions). For adolescents recruited in hospital settings, clinical information was also provided by the nutritionist, and only this source of information was considered. Each adolescent’s and parent’s BMI was calculated using the formula weight/[height]^2^, with weight (kg) and height (m) values. For adolescents, BMI z-scores (zBMI) were calculated according to the recommended WHO Child Growth Standards (2006) using WHO Anthro software provided by the WHO (2010).

#### Mindful Parenting

Parents’ mindful parenting skills were assessed with the Portuguese version of the IMP Scale (Duncan, 2007; Moreira and Canavarro, 2017b). The Portuguese version contains 29 items rated on a five-point Likert response scale ranging from 1 (*never true*) to 5 (*always true*), with higher scores indicating higher levels of mindful parenting. This self-report questionnaire includes five subscales: Listening with Full Attention (e.g., “I rush through activities with my child without being really attentive to him/her”), Emotional Awareness of the Child (e.g., “I notice how changes in my child’s mood affect my mood”), Self-Regulation in Parenting (e.g., “I often react too quickly to what my child says or does”), Non-judgmental Acceptance of Parental Functioning (e.g., “I listen carefully to my child’s ideas even when I disagree with them”), and Compassion for the Child (e.g., “I am kind to my child when he/she is upset”). Both the original and the Portuguese versions have shown reliability and other adequate psychometric properties (Duncan, 2007; Moreira and Canavarro, 2017b). In this sample, Cronbach’s alpha ranged between 0.60 (Non-judgmental Acceptance of Parental Functioning) and 0.80 (Compassion for the Child).

#### Self-Compassion

The Portuguese short form of the Self-Compassion Scale (SCS-SF) was used to measure adolescent’s self-compassion skills (Raes et al., 2011; Castilho et al., 2015). The SCS-SF is a valid and reliable instrument with good psychometric proprieties to measure self-compassion in adolescent samples (Raes et al., 2011; Castilho et al., 2015). The short version includes 12 items (e.g., “I try to be understanding and patient toward those aspects of my personality I don’t like”) answered on a five-point Likert response scale ranging from 1 (*almost never*) to 5 (*almost always*). This instrument measures the six components of self-compassion (self-kindness, self-judgment, common humanity, isolation, mindfulness, and overidentification) and provides a total score for self-compassion, with higher scores reflecting higher self-compassion. In the current study, only the total score for self-compassion was used, and Cronbach’s alpha was 0.75.

#### Body Shame

The body shame subscale of the Experience of Shame Scale (ESS; Andrews et al., 2002; Rodrigues, 2013) was used to assess the intensity with which adolescents have experienced cognitive and behavioral components of body shame in the last 3 months. Although originally developed to be used with adults, this subscale has also been used among adolescents (Moreira et al., 2015) and was validated in the Portuguese population in a sample of adolescents with adequate reliability and validity (Rodrigues, 2013). This subscale has four items (e.g., “Have you avoided looking at yourself in the mirror?”) rated on a four-point Likert response scale ranging from 1 (*not at all*) to 4 (*very much*), with higher scores indicating higher levels of body shame. In the present study, Cronbach’s alpha was 0.84.

#### Emotional Eating

Adolescents’ emotional eating was assessed using the Portuguese version of the Emotional Eating subscale of the Dutch Eating Behavior Questionnaire (DEBQ; van Strien et al., 1986; Viana and Sinde, 2008). This unidimensional instrument assesses the desire to eat under different emotional states (e.g., irritated, depressed, lonely, frightened, and disappointed) with 13 items (“Do you have a desire to eat when feeling lonely?”) rated with a five-point response scale ranging from 0 (*never*) to 4 (*very often*). The DEBQ was originally intended for adults and adolescents, and the original and Portuguese versions have shown good factorial validity and reliability (van Strien et al., 1986; Viana and Sinde, 2008). Higher scores indicate higher levels of emotional eating. In this study, Cronbach’s α was 0.92.

### Data Analyses

Data analyses were conducted using the Statistical Package for the Social Sciences (SPSS Version 22.0; IBM SPSS, Armonk, NY, United States) and AMOS 22 (IBM^®^ SPSS^®^ AMOS™ Version 22.0; IBM Corporation, Meadville, PA, United States).

Descriptive statistics were computed for all sociodemographic, clinical, and study variables. Differences in the study variables as a function of the adolescent’s weight group (i.e., adolescents with normal weight vs. adolescents with overweight/obesity not undergoing nutritional treatment vs. adolescents with overweight/obesity undergoing nutritional treatment) were analyzed with ANOVAs. Pearson correlations between the study variables and between the study variables and parents’ and adolescents’ sociodemographic and clinical variables were determined to identify possible covariates to introduce into the model. Cohen’s guidelines were used to describe effect sizes of the correlations (i.e., small for correlations around 0.10, medium for those near 0.30, and large for correlations at 0.50 or higher; Cohen, 1988).

To examine whether mindful parenting [independent variable (IV)] was associated with emotional eating [dependent variable (DV)] through self-compassion skills [mediator 1 (M_1_)] and body shame [mediator 2 (M_2_)], we tested a path model using the maximum likelihood estimation method. Sociodemographic and/or clinical variables were entered as covariates if they were significantly correlated with the mediators or the DV. Criteria for adequate and good fit between the hypothesized model and the observed data were CFI and TLI values ≥ 0.90 and ≥ 0.95, RMSEA values ≤ 0.08 and ≤ 0.06, and SRMR values ≤ 0.10 and ≤ 0.08, respectively (Browne and Cudeck, 1993; Hu and Bentler, 1999). Indirect effects were estimated using bootstrap resampling procedures with 2000 samples and a 90% bias-corrected confidence interval (BC90% CI). Multigroup analyses were performed to test the structural invariance of the path model across the stages of adolescence (early, ages 12–14, vs. middle/late adolescence, ages 15–18; Spano, 2004), gender (girls vs. boys), weight groups (normal weight vs. overweight/obesity not undergoing nutritional treatment vs. overweight/obesity undergoing nutritional treatment), and parental gender (father vs. mother) and weight (normal weight vs. overweight/obesity). Adolescents in the middle (15–16 years of age; *n* = 164; 69.2%) and late (17–21 years of age, *n* = 73; 30.8%) stages of adolescence were grouped in the same category (Spano, 2004). Each multigroup analysis compared the baseline or unconstrained model (i.e., configural invariance model, which is a model without equality constraints on parameters) with a model in which structural weights were controlled to be equal across groups. The path model was considered to be invariant across groups when a non-significant chi-square difference (Δχ^2^) was found between the constrained and unconstrained models. Secondary analyses were performed to explore the direct and indirect effects in the path model considering all the dimensions of mindful parenting. Specific indirect effects were estimated using an AMOS user-defined estimand. The empirical power tables proposed by Fritz and Mackinnon (2007) for mediation models suggest that the sample size for this study is sufficient to find a mediation effect, including small to medium a and b paths (0.26) with a power of 0.80.

## Results

### Preliminary Analyses

Differences in the study variables between weight groups (i.e., dyads including adolescents with normal weight, overweight/obesity not undergoing nutritional treatment, and overweight/obesity undergoing nutritional treatment) are shown in Table 2. No significant differences were found for mindful parenting (*p* = 0.397), self-compassion (*p* = 0.070), and emotional eating (*p* = 0.161). In contrast, significant differences were found for body shame, with adolescents with overweight/obesity undergoing nutritional treatment presenting higher levels of body shame than those not undergoing nutritional treatment (*p* = 0.021) and those with normal weight (*p* < 0.001). In addition, adolescents with overweight/obesity not undergoing nutritional treatment presented higher levels of body shame than did adolescents with normal weight (*p* < 0.001). Despite this difference in body shame, the three groups were analyzed together in the subsequent analyses.

**Table 2:** Descriptive statistics, differences between weight groups and correlations between study, sociodemographic, and clinical variables.

	Descriptive statistics			Group differences		Correlations between study variables	Correlations between study variables and sociodemographic and clinical variables											
	
									Adolescents				Parents					
	
	*M*	*SD*	Range	*F*	*η_p_*^2^	1	2	3	Age	Gender	zBMI	Presence of health conditions	Age	Gender	Education level	Area of residence	Cohabitation status	BMI
1. Mindful Parenting	106.99	12.30	56.00–137.00	0.93	0.003	–			0.01	−0.04	−0.03	0.02	0.01	0.05	0.08	−0.02	−0.02	−0.09^∗^
2. Adolescents’ Self-Compassion	3.23	0.61	1.08–5.00	2.67	0.009	0.18^∗∗^	–		−0.13^∗∗^	−0.05	−0.12^∗∗^	0.00	−0.01	−0.04	0.07	−0.03	0.02	−0.05
3. Adolescents’ Body Shame	6.77	2.81	4.00–16.00	34.16^∗∗∗^	0.107	−0.09^∗^	−0.48^∗∗^	–	0.20^∗∗^	0.25^∗∗^	0.34^∗∗^	0.17^∗∗^	0.03	0.20^∗∗^	−0.02	−0.07	−0.09^∗^	0.14^∗∗^
4. Adolescents’ Emotional Eating	11.68	9.65	0.00–52.00	1.83	0.006	−0.05	−0.28^∗∗^	0.34^∗∗^	0.10^∗^	0.15^∗∗^	0.08^∗^	0.06	0.00	0.04	−0.10^∗^	−0.05	−0.01	0.04

*Correlations were performed in the total sample. Gender: 0 = male, 1 = female; presence of health conditions: 0 = no, 1 = yes; education level: 0 = basic or secondary education, 1 = graduate or post-graduate education; area of residence: 0 = urban, 1 = rural; cohabitation status: 0 = not living with a partner, 1 = living with a partner. ^∗^p < 0.05; ^∗∗^p < 0.01; ^∗∗∗^p < 0.001.*

### Correlations Between Study Variables and Between Study, Sociodemographic, and Clinical Variables

The descriptive statistics for the study variables and bivariate correlations between the study variables and between the study variables and the sociodemographic and clinical characteristics of adolescents and their parents are presented in Table 2. Positive small to medium correlations were found between mindful parenting and self-compassion and between body shame and emotional eating. Negative small to large correlations were found between mindful parenting and body shame, between self-compassion and body shame, and between self-compassion and emotional eating.

Positive and negative small to medium correlations were found between the study variables and sociodemographic and clinical characteristics (Table 2). Adolescents’ age, gender, zBMI, and presence of health conditions, as well as parents’ gender, education level, cohabitation status, and BMI, were introduced as covariates in the path model.

### Indirect Effect of Mindful Parenting on Adolescents’ Emotional Eating Through Adolescents’ Self-Compassion and Body Shame

**FIGURE 1:**
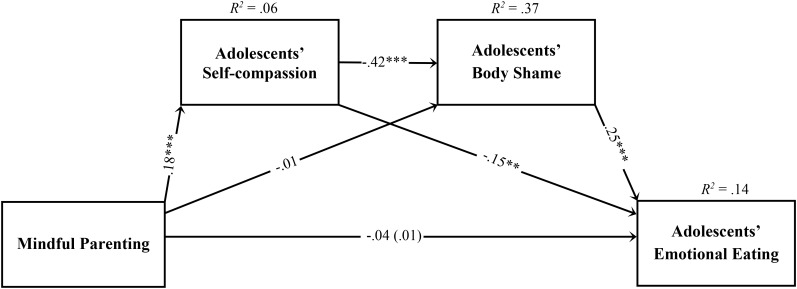
Statistical diagram of the path model estimating the indirect effects of mindful parenting on adolescents’ emotional eating, through adolescents’ self-compassion skills and adolescents’ body shame. Path values represent standardized regression coefficients. The values outside the parentheses represent the total effects and the values in parentheses represent the direct effects of mindful parenting on adolescents’ emotional eating after the inclusion of the mediators. For simplicity, measurement error terms and the covariates are not shown. ^∗∗^*p* < 0.01; ^∗∗∗^*p* < 0.001.

The baseline model with the total score of mindful parenting failed to present a good fit to the data (χ^2^(47) = 286.06, *p* < 0.001; CFI = 0.618; TLI = 0.463; SRMR = 0.088; RMSEA = 0.094, *p* < 0.001; 90% CI = [0.08, 0.11]). Therefore, we examined modification indices, which suggested that the residuals belonging to some of the covariates might be correlated and were performed individually, and the model re-estimated in sequential steps as follows: adolescent’s BMI and parent’s gender, adolescent’s zBMI and parent’s BMI, adolescent’s presence of health conditions and parent’s gender, adolescent’s presence of health conditions and zBMI. The respecified path model presented a good fit to the data, χ^2^(43) = 78.49, *p* < 0.001; CFI = 0.943; TLI = 0.913; SRMR = 0.047; RMSEA = 0.038, *p* = 0.931; 90% CI = [0.02, 0.05] and explained 14% of the adolescents’ emotional eating variance (Figure 1). The difference between the first and final model was significant, Δχ^2^(4) = 207.57, *p* < 0.001, suggesting that the respecified model presented a significantly better fit to the data than did the original model.

As presented in Figure 1, the total and direct effects of mindful parenting skills on adolescents’ emotional eating were non-significant. Moreover, significant indirect effects were found for the association between mindful parenting and adolescents’ body shame through adolescents’ self-compassion skills (*b* = -0.075, *p* = 0.001, 90% CI [-0.106, -0.048]), for the association between adolescents’ self-compassion skills and emotional eating through body shame (*b* = -0.107, *p* = 0.001, 90% CI [-0.145, -0.072]), and for the association between mindful parenting and emotional eating through the two mediators sequentially (*b* = -0.015, *p* = 0.001, 90% CI [-0.023, -0.009]) and through self-compassion only (*b* = -0.021, *p* = 0.002, 90% CI [-0.037, -0.009]). The indirect effect of mindful parenting on emotional eating through body shame only was non-significant (*b* = -0.001, *p* = 0.787, 90% CI [-0.013, 0.010]).

### Invariance Analyses

Multigroup analyses were performed to test the structural invariance of the path model across adolescents’ stage of adolescence, gender, and weight group and parents’ gender and weight. In each model, the variable under study was not introduced as a covariate. The path model was invariant across the two stages of adolescence, across adolescents’ weight groups, across mothers and fathers, and across parents’ weight groups but not across adolescents’ gender (Table 3).

**Table 3:** Invariance analyses.

	Constrained model					Unconstrained model					Δχ^2^	
	χ^2^ (DF)	CFI	TLI	SRMR	RMSEA [CI]	χ^2^ (DF)	CFI	TLI	SRMR	RMSEA [CI]	Δχ^2^ (DF)	*p*
Stage of adolescence	100.74 (86)	0.974	0.967	0.048	0.017 [0.00, 0.03]	82.78 (70)	0.977	0.964	0.045	0.018 [0.00, 0.03]	17.96 (16)	0.326
Adolescent’s gender	125.57 (85)^∗∗^	0.928	0.906	0.049	0.029 [0.02, 0.04]	96.07 (68)^∗^	0.950	0.919	0.030	0.040 [0.01, 0.04]	29.50 (17)	0.030
Adolescent’s weight group	198.59 (146)^∗∗^	0.868	0.985	0.056	0.025 [0.02, 0.03]	157.53 (114)^∗∗^	0.891	0.842	0.054	0.026 [0.02, 0.04]	41.06 (32)	0.131
Parent’s gender	132.01 (88)^∗∗^	0.906	0.882	0.093	0.030 [0.02, 0.04]	104.39 (70)^∗∗^	0.926	0.884	0.082	0.029 [0.02, 0.04]	27.63 (18)	0.068
Parent’s BMI	98.69 (86)	0.977	0.970	0.052	0.016 [0.00, 0.03]	82.64 (68)	0.973	0.956	0.047	0.019 [0.00, 0.03]	16.05 (18)	0.589

*^∗^p < 0.05; ^∗∗^p < 0.01; ^∗∗∗^p < 0.001.*

To identify which paths accounted for the non-invariance between adolescents’ gender groups, we investigated the critical ratios for differences between parameters. There were non-invariant associations between mindful parenting and adolescents’ self-compassion (boys: β = 0.090, *p* = 0.162; girls: β = 0.221, *p* < 0.001) and between adolescents’ self-compassion and adolescents’ body shame (boys: β = -0.251, *p* < 0.001; girls: β = -0.505, *p* < 0.001). Therefore, the relationship between mindful parenting and adolescents’ self-compassion was significant for girls only, and the relationship between adolescents’ self-compassion and adolescents’ body shame was stronger for girls. Examining the differences between the unconstrained model and six models in which the structural weight of a single path was fixed to be equal across groups revealed significant differences between the unconstrained model and the model in which the path linking mindful parenting and adolescents’ self-compassion was constrained, Δχ^2^(1) = 4.85, *p* = 0.028, and the model in which the path linking adolescents’ self-compassion and adolescents’ body shame was constrained, Δχ^2^(1) = 8.23, *p* = 0.004, supporting the differences suggested by the examination of critical ratios. Finally, we analyzed the indirect effects in each group to verify which indirect effects varied. The indirect effects between mindful parenting and adolescents’ body shame through self-compassion (boys: *b* = -0.02, *p* = 0.102, 90% CI = [-0.05, 0.00]; girls: *b* = -0.11, *p* = 0.001, 90% CI = [-0.16, -0.07]) and between mindful parenting and emotional eating through self-compassion followed by body shame (boys: *b* = -0.01, *p* = 0.089, 90% CI = [-0.01, 0.00]; girls: *b* = -0.02, *p* = 0.002, 90% CI = [-0.03, -0.01]) and through self-compassion only (boys: *b* = -0.01, *p* = 0.150, 90% CI = [-0.02, 0.00]; girls: *b* = -0.03, *p* = 0.001, 90% CI = [-0.07, -0.02]) were significant for girls only. The indirect effect between self-compassion and emotional eating through body shame was significant for both groups.

### Exploratory Analyses of the Role of Mindful Parenting Dimensions

**FIGURE 2:**
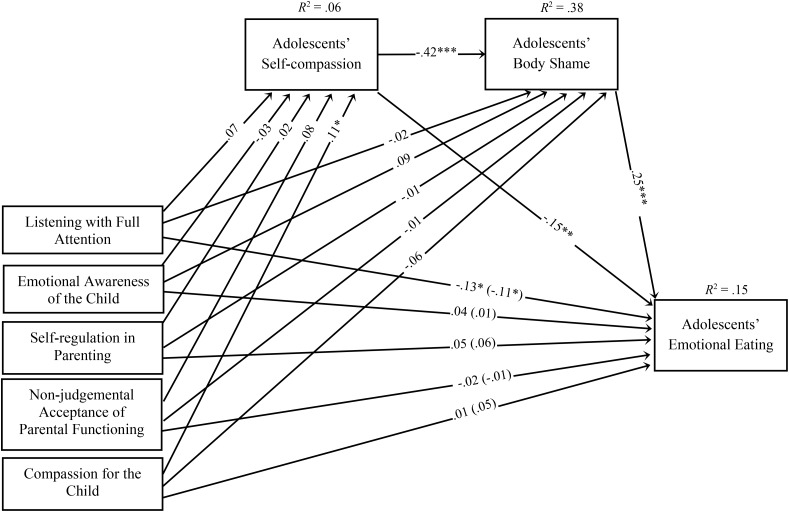
Path model examining the associations between the five dimensions of mindful parenting on adolescents’ emotional eating through adolescents’ self-compassion skills and adolescents’ body shame. Path values represent standardized regression coefficients. The values outside the parentheses represent the total effects and the values in parentheses represent the direct effects of the mindful parenting dimensions on adolescents’ emotional eating after the inclusion of the mediators. For simplicity, measurement error terms and covariates are not shown. ^∗^*p* < 0.05; ^∗∗^*p* < 0.01; ^∗∗∗^*p* < 0.001.

Secondary analyses were performed to explore the direct and indirect effects of mindful parenting dimensions on adolescents’ emotional eating, and the same covariates of the model with the total score were entered and correlated with each other (Figure 2). The model demonstrated a good fit to the data (χ^2^(75) = 155.05, *p* < 0.001; CFI = 0.944; TLI = 0.910; SRMR = 0.048; RMSEA = 0.043, *p* = 0.872; 90% CI = [0.03, 0.05]) and explained 15% of the variance in adolescents’ emotional eating. Direct effects are presented in Figure 2, and indirect effects are presented in Table 4. Significant direct effects were found between compassion for the child and self-compassion, between listening with full attention and emotional eating, between self-compassion and body shame, between self-compassion and emotional eating, and between body shame and emotional eating. Moreover, several significant indirect effects were found, namely, between compassion for the child and body shame through self-compassion, between self-compassion and emotional eating through body shame, and between compassion for the child and emotional eating through self-compassion only and through self-compassion skills followed by body shame.

**Table 4:** Indirect and specific indirect effects of mindful parenting dimensions on adolescents’ emotional eating through adolescents’ self-compassion and body shame.

	Unstandardized coefficients	Standardized coefficients	*p*-value	BC90%CI
				Lower/upper
*Indirect effects*		
LFA → Self-compassion → Body Shame	−0.025	−0.028	0.208	−0.064/0.008
EAC → Self-compassion → Body Shame	0.016	0.013	0.609	−0.027/0.051
SR → Self-compassion → Body Shame	−0.049	−0.009	0.650	−0.050/0.026
NJAPF → Self-compassion → Body Shame	−0.161	−0.033	0.112	−0.069/0.001
CC → Self-compassion → Body Shame	−0.219	−0.048	0.042	−0.085/−0.008
LFA → Self-compassion → Body Shame → Emotional Eating	−0.067	−0.022	0.179	−0.054/0.004
EAC → Self-compassion → Body Shame → Emotional Eating	0.129	0.030	0.091	0.001/0.063
SR → Self-compassion → Body Shame → Emotional Eating	−0.161	−0.009	0.577	−0.040/0.018
NJAPF → Self-compassion → Body Shame → Emotional Eating	−0.343	−0.020	0.172	−0.046/0.005
CC → Self-compassion → Body Shame → Emotional Eating	−0.713	−0.045	0.014	−0.081/−0.015
Self-compassion → Body Shame → Emotional Eating	−1.675	−0.106	0.001	−0.145/−0.072
*Specific indirect effects*		
CC → Self-compassion → Emotional Eating	−0.265		0.026	−0.605/−0.060
CC → Body Shame → Emotional Eating	−0.256		0.194	−0.684/0.060
CC → Self-compassion → Body Shame → Emotional Eating	−0.192		0.030	−0.367/−0.045

*Specific indirect effects were only estimated for significant general indirect effects. LFA, listening with full attention; EAC, emotional awareness of the child; SR, self-regulation in parenting; NJAPF, non-judgmental acceptance of parental functioning; CC, compassion for the child; BC90%CI, 90% bias-corrected and accelerated confidence interval.*

## Discussion

This study explored an integrative model suggesting that higher levels of mindful parenting skills were associated with lower levels of adolescents’ emotional eating through higher levels of adolescents’ self-compassion skills in isolation or followed by lower levels of body shame, but only among adolescent girls, regardless of their weight. Therefore, these findings suggest two sequences of mechanisms through which a mindful parenting approach might be associated with adolescents’ emotional eating behavior. Mindful parenting may allow the development of self-compassion skills in adolescents, which may reduce emotional eating, and these self-compassion skills may allow adolescents to accept their body shape and appearance, thus preventing engagement in eating behaviors to compensate for negative emotional states. Additionally, the mindful parenting dimension of compassion for the child was the only dimension that was indirectly associated with emotional eating. Our findings also suggest that both adolescent boys and girls with greater self-compassion skills engaged less in emotional eating behaviors because they had lower levels of body shame.

As suggested in previous studies, mindful parenting might be an ideal ground for the development of important psychological resource in adolescents, such as self-compassion (Moreira et al., 2018). Parents with higher levels of mindful parenting foster positive and secure parent–child relationships based on a warm, compassionate, acceptant, and respectful mentality that in turn promotes adolescents’ well-being (Medeiros et al., 2016) and self-compassion skills (Moreira et al., 2018). Moreover, these parents are generally more likely to adopt adaptive coping strategies when facing difficult thoughts and emotions, as they tend to have higher levels of self-compassion and dispositional mindfulness (Gouveia et al., 2016; Moreira et al., 2016). Therefore, a child with parents with higher levels of mindful parenting may develop self-compassion skills by both observing and modeling how their parents generally relate to themselves in difficult situations (i.e., observational learning) and how parents relate to their children and their emotions (i.e., positive family experiences; Neff and McGehee, 2010; Moreira et al., 2016). Both of these experiences may stimulate a self-compassionate inner dialog in the child that is particularly important when experiencing negative emotions. Therefore, this study provides further support to consider self-compassion as a psychological mechanism through which mindful parenting influences adolescents’ psychological outcomes.

By developing a stance of self-compassion and self-kindness toward themselves, adolescents may engage less in self-judgments, overidentification with internal states, or isolation in times of suffering but may instead develop an attitude of self-kindness when facing their own suffering by being aware of it and recognizing that all human beings suffer (Neff, 2003, 2009). Therefore, these adolescents will be more capable of addressing the challenges of this developmental stage, such as the challenges associated with fitting the ideal body image perpetuated by society (i.e., thinness schema; e.g., Latner and Stunkard, 2003; Puhl and Latner, 2007). With body shame considered to be a self-oriented emotion based on ruminative and self-critical thoughts and emotions (Cheung et al., 2004), having higher levels of self-compassion may prevent the cycle of negative self-evaluations of one’s body and subsequent generalizations to more global negative feelings about the self. These findings are supported by previous studies that have found a significant negative association between mindfulness skills and body shame in adolescents with overweight/obesity (Moreira and Canavarro, 2017a) and have suggested that the experience of body shame is inversely associated with a present-centered awareness and a compassionate and non-judgmental stance (Woods and Proeve, 2014). Because mindful awareness and a compassionate stance are intrinsically associated, developing these psychological resources may help adolescents to distance themselves from self-ruminative thoughts about their appearance and from experiencing shame in this domain.

In addition, as suggested by the results of this study and in accordance with previous studies (e.g., Mustapic et al., 2015; Iannaccone et al., 2016), when this cycle of negative self-oriented emotions based on appearance is interrupted, adolescents are less likely to engage in emotional eating behaviors. For instance, body shame generates negative thoughts and emotions about one’s body and oneself in general that often have a critical tone, which may increase the urge to engage in compensatory behaviors to alleviate these negative emotions. This maladaptive cycle of emotion regulation may foster certain eating behaviors as a way to address negative (and unpleasant) emotions, that is, to eat when negative emotions are inadequately regulated. Moreover, the results of this study found a significant negative association between self-compassion skills and emotional eating. Although similar results have already been found among adult women with normal weight and obesity (e.g., Ferreira et al., 2013; Braun et al., 2016; Rahimi-Ardabili et al., 2018), to our knowledge, this study is the first to demonstrate this association among adolescents with different weights. Related to the previous result, having higher levels of self-compassion skills, which is also considered a positive psychological resource, may allow negative emotions to be regulated with a kind and non-judgmental perspective instead of with overidentification and a critical attitude (e.g., Adams and Leary, 2007; Mantzios and Wilson, 2014). Therefore, developing self-compassion decreases engagement in disordered eating behaviors such as emotional eating by interrupting the maladaptive cycle of emotion regulation.

We also found that both adolescent boys and girls with higher levels of self-compassion had lower levels of emotional eating and that this association was mediated by lower levels of body shame. This novel result suggests an interrelationship among these constructs and a sequence of mechanisms that may generate emotional eating behaviors among adolescents. Although these results highlight the role that self-compassion skills and body shame may play in adolescents’ eating behavior, future longitudinal studies should ascertain whether adolescents’ self-compassion can lead to lower levels of body shame or whether higher body shame can lead to lower levels of self-compassion skills, which can in turn trigger emotional eating behaviors.

Secondary analyses with all the mindful parenting dimensions showed that compassion for the child was the only significant dimension indirectly associated with emotional eating. Being compassionate, kind, and sensible with respect to a child’s needs seems to be particularly important in promoting the development of self-compassion skills in adolescents, which may in turn protect adolescents from experiencing body shame and engage in eating behaviors to compensate for these negative emotional states. These novel findings are supported by the theoretical background and recent studies. For instance, Moreira et al. (2018) found that being a compassionate parent may foster a more secure relationship between the parent and the child, which in turn promotes the development of adolescents’ self-compassion skills. In the present study, being a compassionate parent was directly associated with adolescents’ self-compassion. In fact, by adopting an attitude of kindness, sensitivity, and responsiveness to the child’s needs, a parent may transmit to the child (both directly and indirectly through their actions) how to adequately relate to oneself, especially when facing difficult situations.

Additionally, we found a negative direct link between listening with full attention to the child and adolescents’ emotional eating. Although this finding warrants further investigation, it suggests that when parents are fully present in parent–child interactions and direct their complete attention to their children, those children may feel more emotionally supported and have less need to engage in emotional compensatory behaviors (e.g., to eat) to alleviate their emotions. In this way, the lack of emotional attention that could be temporarily satisfied by food could be reduced since the parents provided the emotional attention the child needed. Although these are tentative explanations, this result indicates that a mindful approach to parenting may influence children’s emotion regulation processes, which may be masked by eating behaviors. Therefore, further investigation is needed to deepen the knowledge of how mindful parenting may influence children’s eating behaviors.

Another relevant finding was that these indirect effects were significant for girls only. As hypothesized, the associations in the model may be more salient among girls than among boys since body image-related issues and disordered eating behaviors are especially common among adolescent girls (Pearson et al., 2012; Bluth et al., 2017). In fact, previous research has shown that compared with girls, boys are not as heavily influenced by the body image ideals perpetuated by society and are not as prone to engage in eating behaviors to compensate for negative emotions (Puhl and Latner, 2007; Bluth et al., 2017).

In contrast to our expectations, the model was invariant across the stages of adolescence, although, as expected, lower levels of self-compassion skills and higher levels of body shame and emotional eating were significantly correlated with the middle/late stage of adolescence. The model was also invariant across weight groups (adolescents with normal weight and with overweight/obesity undergoing or not undergoing nutritional treatment). Moreover, in contrast to our expectations, we found no differences between weight groups for self-compassion skills and emotional eating; however, a negative correlation was found between zBMI and self-compassion skills, and positive correlations were found between zBMI and body shame and between zBMI and emotional eating. Significant differences between weight groups were found for body shame only, as adolescents with overweight/obesity who were undergoing nutritional treatment presented higher levels of body shame than did adolescents in other groups. In addition, adolescents with overweight/obesity who were not undergoing nutritional treatment presented higher levels of body shame than did adolescents with normal weight; these findings are in accordance with previous studies (Goossens et al., 2009). Therefore, a better understanding of the role of the stage of adolescence and weight group might provide important insights into which adolescents may benefit more from specific interventions, such as interventions based on self-compassion.

Some limitations of this study must be noted. First, the cross-sectional design of the study prevents the establishment of causal relationships; therefore, alternative models may be hypothesized. For instance, self-compassion skills could mediate the association between body shame and emotional eating behaviors in adolescents since this psychological resource is aimed at alleviating negative emotions. Nevertheless, future studies with longitudinal designs may ascertain the direction of these associations or identify which of them may be more beneficial at the clinical level. Second, although this study comprised a large sample, it was collected from only three public schools and three hospitals in the central region of Portugal, and most parents were mothers, had overweight/obesity, were living with a partner, had completed basic or secondary education, and lived in rural areas; these characteristics compromise the representativeness of the sample and the generalization of the results to parents from different sociodemographic backgrounds. Third, two different procedures were used to collect anthropometric data from adolescents: for adolescents undergoing nutritional treatment, weight and height were objectively measured by the nutritionist, whereas for adolescents not undergoing nutritional treatment, weight and height were subjectively measured by self-reports. Future studies should overcome this limitation by using the same calibrated balance with all participants since youths may not accurately self-report their weight and height (Brener et al., 2003; Tokmakidis et al., 2007). Moreover, it would have been interesting to monitor nutritional treatment and weight over time and to understand the physical activity habits of the adolescents. Fourth, only self-report measures were used, which can compromise the validity of the results because participants may be influenced by social desirability and not reliably report their inner states. Fifth, the Emotional Eating subscale of the DEBQ measures the urge to eat rather than the actual eating behavior. Future studies should use more proximal instruments that can measure the frequency of real eating episodes under different emotional states. Sixth, in this sample, Cronbach’s alphas between 0.60 and 0.70 were obtained for three of the mindful parenting subscales (Emotional Awareness of the Child, Self-regulation in Parenting, and Non-judgmental Acceptance of Parental Functioning). Nevertheless, some authors agree that Cronbach’s alpha values above 0.60 are adequate, particularly in exploratory and psychology research, even though the generally acceptable lower limit is 0.70 (Aron and Aron, 1999).

Despite these limitations, this study has important strengths. It provides preliminary evidence for the benefits of adopting a mindful parenting approach in the context of adolescents’ eating behavior, and it improves our understanding of the mechanisms explaining why this parenting approach is associated with adolescents’ emotional eating. Specifically, the current study proposes an innovative comprehensive model of the sequence of mechanisms underlying the relationship between mindful parenting and emotional eating in adolescents with different weights, highlighting the complexity and the interrelationship between parent and adolescent variables. This study innovatively suggests that mindful parenting, particularly compassion for the child, plays an important role in conveying a caring and compassionate attitude to the child when facing difficult emotions, protecting the child against experiencing higher levels of body shame, and engaging in emotional eating behaviors to compensate for these negative emotional states. By studying these associations in a sample of adolescents with different weights, this study provides a novel and promising approach to the study of adolescents’ eating behaviors. For instance, by developing a mindful posture in parenting, parents may help adolescents develop self-compassion skills, which may be very beneficial for adolescents’ psychological adjustment and eating behavior. Nevertheless, the cross-sectional design of this study accentuates the need for future longitudinal studies and randomized controlled trials on mindful parenting-based interventions to better understand the presumed positive impact of this parental approach.

These results also provide further support to consider the role of gender in these associations, since the model was significant for adolescent girls only. Therefore, girls would probably benefit more from an intervention aimed at diminishing body shame and emotional eating issues. According to the results of this study, such an intervention could include self-compassion training for adolescents and a mindful parenting-based intervention, with a special focus on the dimension of compassion for the child but also on the dimension of listening with full attention to the child. Additionally, the associations between the variables in the model are invariant across adolescents’ weight groups, which suggests that the regulation of negative emotions about one’s body does not depend on the weight. Nevertheless, the role of adolescents’ gender and weight status has received little attention with respect to mindful parenting, and future studies should further explore it.

## Conclusion

Emotions can have a substantial impact on eating behavior. Therefore, it is necessary to shift the focus of disordered eating behavior treatment from only dietary-based programs to more complete and comprehensive approaches. This study provides preliminary and novel support to consider the beneficial role of both mindful parenting and self-compassion skills in adolescents struggling with feelings of body shame and engaging in emotional eating behaviors. Therefore, a broader approach that includes both adolescents and their parents and that targets both the eating behaviors and the emotional processes behind those behaviors may have more long-term results. Considering the importance that adolescents place on their bodies and how it may influence their psychological adjustment and eating behavior, developing a compassionate posture when facing negative emotions might facilitate, from an early age, a healthier relationship with their emotions and bodies and ultimately with food. As parents are significant figures in adolescents’ lives, they may play a determinant role in promoting self-compassion skills in their children. The clinical implications are promising, but future studies with more robust methodologies are certainly needed.

## Ethics Statement

This study was carried out in accordance with the ethical standards of the institutional and/or national research committee and with the 1964 Helsinki declaration and its later amendments or comparable ethical standards with written informed consent from all subjects. All subjects gave written informed consent in accordance with the Declaration of Helsinki of 1964. The protocol was approved by the Portuguese Data Protection Authority, the Ethics Committee of the Faculty of Psychology and Educational Sciences of the University of Coimbra, the Board of Directors of each school unit, and the Ethics Committee and the Board of Directors of each hospital.

## Author Contributions

MG designed and executed the study, assisted with the data analyses, and wrote the paper. MC collaborated in the writing and editing of the final manuscript. HM collaborated with the design, data analyses and writing of the study. All authors approved the final version of the manuscript for submission.

## Conflict of Interest Statement

The authors declare that the research was conducted in the absence of any commercial or financial relationships that could be construed as a potential conflict of interest. The reviewer AU and handling Editor declared their shared affiliation at the time of the review.
